# New bio-nanocomposites based on iron oxides and polysaccharides applied to oxidation and alkylation reactions

**DOI:** 10.3762/bjoc.13.194

**Published:** 2017-09-21

**Authors:** Daily Rodríguez-Padrón, Alina M Balu, Antonio A Romero, Rafael Luque

**Affiliations:** 1 Departamento de Química Orgánica, Grupo FQM-383, Universidad de Cordoba, Campus de Rabanales, Edificio Marie Curie (C-3), Ctra Nnal IV-A, Km 396, E14014, Cordoba, Spain

**Keywords:** alkylation, benzyl alcohol, benzyl chloride, iron oxide, mechanochemistry, microwave-assisted oxidation, polysaccharide, toluene

## Abstract

Polysaccharides from natural sources and iron precursors were applied to develop new bio-nanocomposites by mechanochemical milling processes. The proposed methodology was demonstrated to be advantageous in comparison with other protocols for the synthesis of iron oxide based nanostructures. Additionally, mechanochemistry has enormous potential from an environmental point-of-view since it is able to reduce solvent issues in chemical syntheses. The catalytic activity of the obtained nanocatalysts was investigated in both the oxidation of benzyl alcohol to benzaldehyde and in the alkylation of toluene with benzyl chloride. The microwave-assisted oxidation of benzyl alcohol reached 45% conversion after 10 min. The conversion of the alkylation of toluene in both microwave-assisted and conventional heating methods was higher than 99% after 3 min and 30 min, respectively. The transformation of benzyl alcohol and toluene into valuable product in both the oxidation and alkylation reaction reveals a potential method for the valorization of lignocellulosic biomass.

## Introduction

Heterogeneous catalysis has played a crucial role in the development of the chemical industry. It has allowed the design of more efficient processes, both in an economical and environmental way, thanks to the higher activity and selectivity of heterogeneous catalysts [1–3]. These systems, in particular, are preferred over the use of catalysts in a homogeneous phase due to the difficulty in separation and recovery of the latter. Heterogeneous catalytic systems, as a priority of research activity in the field of green chemistry, open up new possibilities for further development of environmentally friendly, catalyzed processes [4]. In this sense, metal oxide nanoparticles have been extensively studied in recent decades because of their high activity, specificity of interaction and advantageous properties including a high surface/volume ratio combined with their small size [5–7]. Moreover, metal oxide nanoparticles have the additional advantage of easy recycling and reuse, which is an essential and desired property in many applications such as catalysis, sensors and even medicine [2,6,8–9]. Our research group has recently prepared different types of metal and metal oxide nanoparticles which have several applications in heterogeneous catalysis [10–14]. Transition metal and metal oxide nanoparticles have been reported to be highly active and selective in several processes, such as redox [15–17], C–C and C–heteroatom couplings [18–19]. In particular, iron oxide nanoparticles have been the object of most research from our group over the past years [10,20–22].

One of the main challenges in the field of catalysis is the preparation of new materials to replace the traditional catalysts quickly, cheaply and efficiently [5]. In this regard, mechanochemical synthesis has become one of the most advantageous and environmentally friendly alternatives compared to the traditional routes [5,23]. This novel approach offers the possibility of a solvent-free process, avoiding environmental problems related to toxicity and the use thereof [24–25]. Moreover, the mechanochemical protocols have potential applicability due to the extreme simplicity, cleanliness, reproducibility and versatility, haven been already demonstrated to be highly useful for the development of a range of advanced nanomaterials including metal-organic frameworks (MOFs), supported metal and metal oxide nanoparticles and nanocomposites with diverse applications in catalysis, sensing, drug delivery and adsorption [25–28]. In addition, mechanochemical protocols have also been employed to functionalize the surfaces of magnetic nanoparticles (MNPs) with monosaccharides [29] and to obtain bio-nanocomposites based on proteins and dopamine (DA)-coated metal oxide MNPs [30–31].

On the other hand, nature has inspired many scientists to innovate and design new materials. The miniaturization and efficiency achieved by entities in nature for energy production, biometabolite, photo-processing and resource maximization has always been an attractive option to imitate based on a fundamental and rational understanding [28,32]. In that sense, polysaccharides extracted from fungal organisms can be used both as nanoparticle carriers and sacrificial templates due to their highly functionalized structure. Although such carbohydrates have been widely reported for the preparation of nanocomposites with a great range of applications, due to their low cytotoxicity and notable biocompatibility and stability [33–37], their catalytic application is still lacking. In addition, these natural products are easily and inexpensively produced by microbes, plants, and animals, and constitute a green alternative to synthetic polymers in the preparation of nanomaterials, in order to ameliorate environmental issues [34]. Therefore, one of the objectives of this work was to investigate the catalytic behavior of nanocomposites based on iron oxide and the polysaccharide S4, obtained from *Lentinus Tigrinus* (PS4).

The most promising feature of such nanoentities based on iron oxide and polysaccharides is the bifunctional, oxidative [20] and acidic nature [21], which in turn can be fine-tuned to design highly active materials for both oxidation and acid catalyzed processes.

Among all the known oxidative transformations, the oxidation of alcohols to ketones and aldehydes have gained a lot of attention for the research community due to its broad range of industrial applications [38–39]. Nonetheless, the scale up of the oxidation reactions has been very restricted due to the use of heavy metals, the limited selectivity for highly functionalized compounds, and the thermal hazards posed [40]. Consequently, catalytic reactions should be further investigated in order to find new alternatives to conventional oxidation methods that require stoichiometric amounts of inorganic oxidants, which are highly toxic and polluting. Aiming to minimize chemical waste in these catalytic processes, the scientific community is moving towards the use of clean oxidants ("green oxidants"), such as molecular oxygen or H_2_O_2_ [39]. Thus, the use of clean oxidants with heterogeneous catalysts such as Fe_2_O_3_ nanoparticles, Ag nanoparticles supported on hydrotalcites, Au nanoparticles supported on metal oxides, and Pd nanoparticles supported on SBA-15 has been developed [41–44]. In this regard, both unsupported “free” iron oxide nanoparticles [45] and supported iron oxide based catalytic systems [46] have been extensity reported to be active, stable and selective catalysts for the oxidation of alcohols with hydrogen peroxide. Specifically, the oxidation of benzyl alcohol to benzaldehyde has generated great interest in order to study the oxidation of substituted benzyl alcohols. Although benzyl alcohol is industrially produced by reduction of benzaldehyde, this aldehyde is considered as the second most important flavoring molecule after vanillin, due to its variety of applications in cosmetics, perfumes, food, dyes, agrochemicals and pharmaceuticals [41]. Regarding the acid-catalyzed processes, aromatic alkylation reactions are among the most versatile and widely investigated reactions which can grant access to a wide range of compounds as important intermediates, fragrances, agrochemicals and pharmaceuticals [47–49]. In this sense, the benzylation of benzene or other aromatic substrates is well-known to be an important step in the preparation of relevant building blocks in organic synthesis, such as diphenylmethane and substituted diphenylmethanes [50]. Therefore, many studies have been focus on the preparation of novel Lewis acid catalysts, such as mesostructured zeolitic materials. In particular, in this study, our research group has focused attention on the alkylation of toluene with benzyl chloride, since is promoted by the presence of Lewis acids such as iron oxides [49].

These two reactions in particular (oxidation and alkylation of benzyl alcohol and toluene, respectively) could find current application in the valorization of lignocellulosic biomass with heterogeneous catalysis.

However, the use of heterogeneous catalysts in the aforementioned reactions usually requires a filtration or centrifugation step to recover the catalyst. In order to simplify the recovery and reuse of the catalytic system, a magnetically separable nanocomposite could represent a breakthrough in the scientific community [51]. Therefore, our research group has focused on the investigation of the aforementioned oxidation and alkylation reactions, using heterogeneous catalysts with magnetic properties.

## Results and Discussion

**Figure 1:**
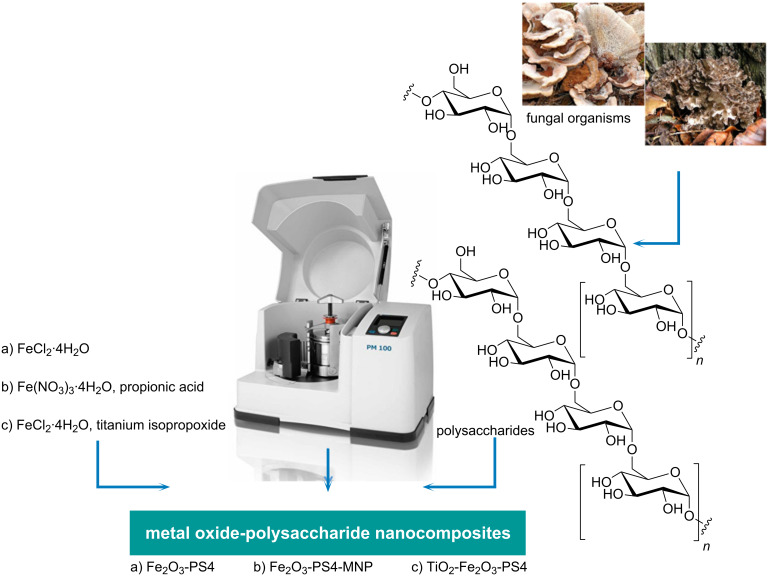
Overview of the preparation of the nanocomposites based on iron oxide and polysaccharide.

In the present study, we prepared and analyzed three different catalysts based on iron oxides and polysaccharides, in particular: iron oxide–polysaccharide 4 magnetic nanoparticles (Fe_2_O_3_-PS4-MNP), iron oxide–polysaccharide 4 (Fe_2_O_3_-PS4) and titanium oxide–iron oxide–polysaccharide 4 (TiO_2_-Fe_2_O_3_-PS4) nanocomposites. The materials were successfully obtained using the proposed solvent-free methodology, which is depicted in Figure 1. The materials were characterized by the techniques presented below. The catalytic activity of these systems has been assessed in the alkylation reaction of toluene with benzyl chloride and the selective oxidation of benzyl alcohol to benzaldehyde.

### X-ray diffraction

The structure and arrangement of the synthesized materials were analyzed by X-ray diffraction. The XRD pattern of the Fe_2_O_3_-PS4 nanomaterial exhibited a series of distinctive diffraction lines that could be correlated to the hematite diffraction pattern. A characteristic broad band in the 20° to 40° range, typical of amorphous materials, was observed in the Fe_2_O_3_-PS4 nanomaterial (Supporting Information File 1, Figure S1A). The X-ray diffraction patterns of the magnetic material showed a mixture of maghemite and hematite phases. In this case, a similar XRD pattern could be in principle associated to magnetite (Fe_3_O_4_) over the maghemite phase, since these two phases are difficult to clearly distinguish by XRD analysis. However, the absence of Fe^2+^ species (see the following XPS analysis) and the reddish color are consistent with a maghemite magnetic phase (Supporting Information File 1, Figure S1B) [51–52]. On the other hand, the crystal structure of the material TiO_2_-Fe_2_O_3_-PS4 turned out to be a mixture of ilmenite and pseudobrookite phases (Supporting Information File 1, Figure S1C).

### X-ray photoelectron spectroscopy

**Figure 2:**
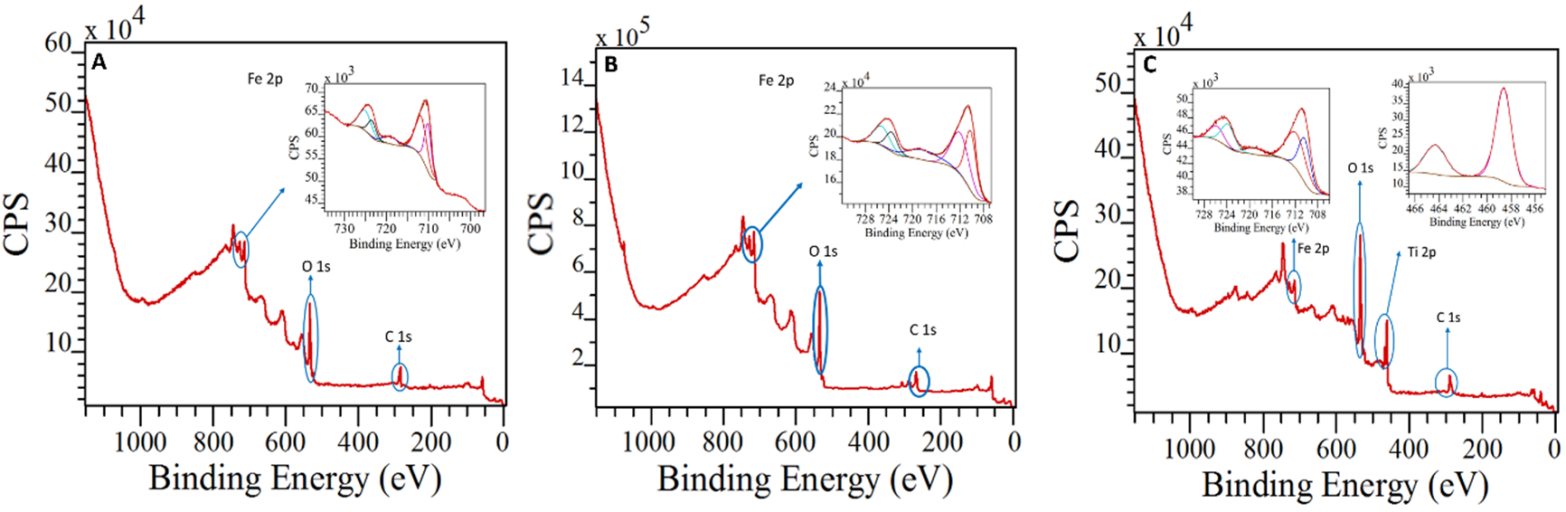
XPS spectra of A: Fe_2_O_3_-PS4, B: Fe_2_O_3_-PS4-MNP and C: TiO_2_-Fe_2_O_3_-PS4 nanohybrids.

X-ray photoelectron spectroscopy (XPS) measurements were consistent with XRD data, where the main peaks were found to correspond to Fe_2_O_3_ species. In the three nanocomposites, the presence of Fe^3+^ species could be also inferred from the Fe 2p_3/2_ and Fe 2p_1/2_ peaks around 710 eV and 725 eV, respectively (Figure 2). These results are in good agreement with previous studies and did not show the characteristic peak associated with the presence of Fe(II) or Fe(0) species in the materials [51–52]. Concerning the TiO_2_-Fe_2_O_3_-PS4 nanocatalyst, the XPS experiments results revealed a band at 462 eV (Ti 2p_3/2_), which confirmed the presence of TiO_2_ on the surface of the nanocomposite (Figure 2C). Additionally, the deconvoluted C 1s XPS spectra of the obtained materials exhibited two different contributions associated to the presence of C–C/C=C and C–O bonds. Also, the O 1s XPS spectra for the Fe_2_O_3_-PS4 and Fe_2_O_3_-PS4-MNP nanomaterials displayed two different peaks attributed to O–C and O–Fe, while for the TiO_2_-Fe_2_O_3_-PS4 nanocomposite contained three contributions related to O–C, O–Ti and O–Fe (see also Supporting Information File 1, Figures S2–S4 for the XPS spectra).

### Nitrogen physisorption

The textural properties of the materials have been studied with N_2_ absorption–desorption isotherms analysis. The TiO_2_-Fe_2_O_3_-PS4 nanomaterial presents a mesoporous structure with a pore size of 20 nm and a surface area of 58 m^2^/g. However, in the other two catalysts, a particular macroporosity was found at *p*/*p*_0_ > 0.98 (isotherms of type III), which are clearly dissimilar to those of conventionally ordered mesoporous materials [53] having a sharp increase in *p*/*p*_0_ from 0.85 to 0.90 (see Supporting Information File 1, Figure S5 for all adsorption–desorption isotherms). Thus, the Fe_2_O_3_-PS4 and the Fe_2_O_3_-PS4-MNP material are macroporous solids with interparticle pores. The surface area was found to be 33 and 6 m^2^/g for Fe_2_O_3_-PS4-MNP and for Fe_2_O_3_-PS4 nanomaterials, respectively. The pore volumes were found to be in the range of 0.30–0.40 mL/g for the three materials (Table 1). The materials exhibited, in general, satisfying surface areas and pore volumes, particularly taking into account their preparation methodology.

**Table 1:** Textural properties of iron oxide/polysaccharide nanohybrids.

Catalyst	TiO_2_-Fe_2_O_3_-PS4	Fe_2_O_3_-PS4-MNP	Fe_2_O_3_-PS4

*S*_BET_ (m^2^/g)	58	33	6
*D*_BJH_ (nm)	20.9	41.2	171.2
*V*_BJH_ (mL/g)	0.32	0.36	0.40

### Electron microscopy

**Figure 3:**
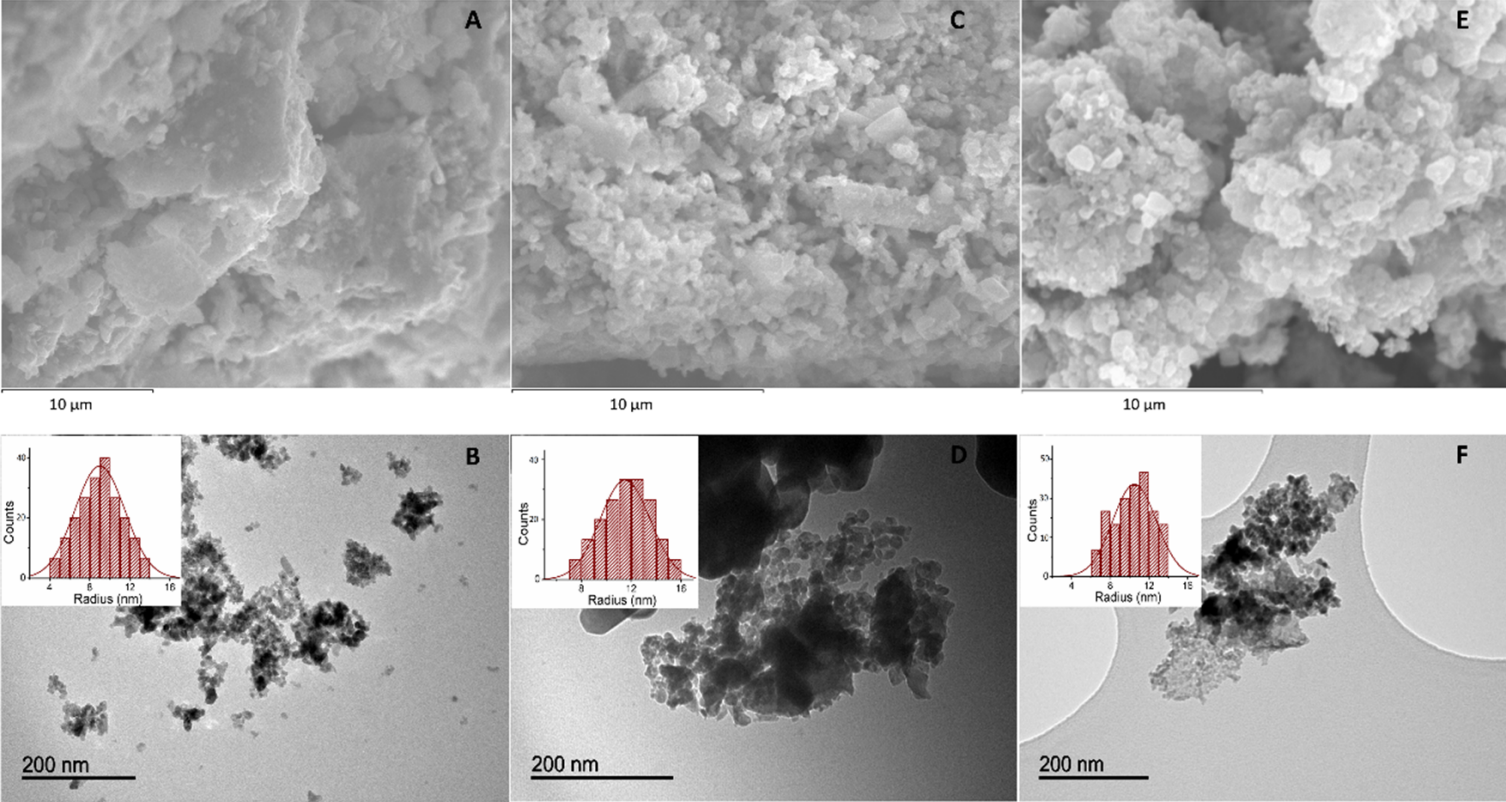
A and B: SEM and TEM images of TiO_2_-Fe_2_O_3_-PS4; C and D: SEM and TEM images of Fe_2_O_3_-PS4. E and F: SEM and TEM images of Fe_2_O_3_-PS4-MNP. Inset: Particle-size distribution of the obtained nanohybrids.

The morphology of the nanomaterials was determined by scanning electron microscopy (SEM) and transmission electron microscopy (TEM). The micrographs show a homogeneous distribution of iron oxide nanoparticles for the three catalysts (Figure 3A,C,E). The analysis of the SEM images reveals the tendency of the constituent particles of the magnetic material to form agglomerates due to their nanometer size (Figure 3E). When these agglomerates are observed at higher magnification, they can be seen as independent particles. The three materials displayed a similar particle-size distribution average of around 9 nm, 12 nm and 10 nm for the TiO_2_-Fe_2_O_3_-PS4, Fe_2_O_3_-PS4 and Fe_2_O_3_-PS4-MNP, respectively (Figure 3B,D,F).

### Diffuse reflectance infrared Fourier transform spectroscopy

**Figure 4:**
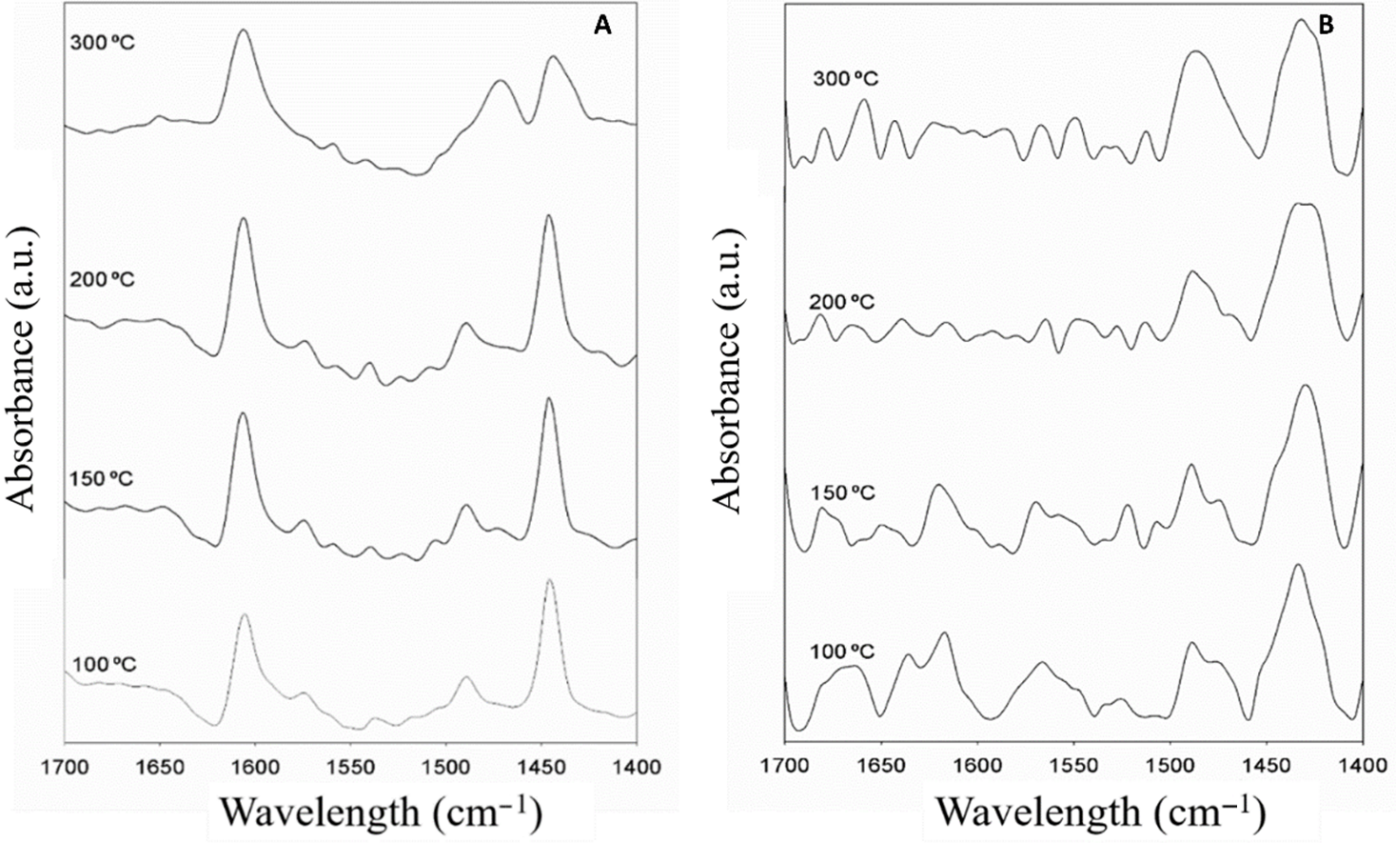
DRIFT spectra of A: TiO_2_-Fe_2_O_3_-PS4 and B: Fe_2_O_3_-PS4-MNP nanohybrids.

The acidic properties of the Fe_2_O_3_-PS4-MNP and TiO_2_-Fe_2_O_3_-PS4 materials were studied by diffuse reflectance infrared Fourier transform spectroscopy (DRIFT) experiments. The TiO_2_-Fe_2_O_3_-PS4 nanocomposite has well-marked acidic characteristics. This can be deduced from the intense and well-defined bands observed at 1449 and 1600 cm^−1^, which can be attributed to Lewis acid centers (Figure 4A). Additionally, in the spectrum of Fe_2_O_3_-PS4-MNP, having bands at 1440 and 1618 cm^−1^, indicates the peculiar Lewis acidity of this material (Figure 4B). Furthermore, in both materials, a band of lesser intensity can be seen around 1490 cm^−1^, which is due to the presence of both Brønsted and Lewis centers.

These materials maintained a remarkable acidity, even at high temperatures (200 and 300 °C) with visible acid centers distinguishable from noise. This behavior has a high value for acid-catalyzed processes such as alkylation. Furthermore, the Fe_2_O_3_-PS4 sample does not show appreciable acidity [43].

### Pyridine (PY) and 2,6-dimethylpyridine (DMPY) titration

The acidic properties of these materials have also been determined by the chromatographic method of pulses. Pyridine, due to low steric hindrance, adsorbs nonspecifically in both types of centers, while dimethylpyridine adsorbs specifically on Brønsted acid centers, due to the high steric hindrance of the methyl groups [54]. It is noticeable that the TiO_2_-Fe_2_O_3_-PS4 catalyst possesses both Lewis and Brønsted acid sites with a more marked Lewis acidity. The Fe_2_O_3_-PS4-MNP material presents instead only Lewis acid sites, while the Fe_2_O_3_-PS4 does not show appreciable acidity to be quantized (Table 2).

**Table 2:** Surface acidity of iron oxide/polysaccharide nanohybrids.

Catalyst	Total acidity PY (µM/g)	Brønsted acidity DMPY (µM/g)	Lewis acidity (µM/g)

TiO_2_-Fe_2_O_3_-PS4	81	25	56
Fe_2_O_3_-PS4-MNP	14	_	14

Acidity measurements from both methodologies (PY DRIFT, PY and DMPY pulse chromatography titration data) were generally in good agreement, supporting the validity of our assumption on DMPY adsorbing selectively on Brønsted acid sites.

### Inductively coupled plasma–mass spectrometry (ICP–MS)

The elemental composition of the TiO_2_-Fe_2_O_3_-PS4 material was determined by ICP–MS. The content of iron and titanium was 38 and 12 wt %, respectively (Table 3). These values corroborate the incorporation of titanium in the material and confirm the results obtained by XPS.

**Table 3:** Elemental composition of the TiO_2_-Fe_2_O_3_-PS4 nanohybrid material.

Element	ICP–MS (wt %)

Ti	12.8
Fe	38.3

### Magnetic susceptibility

The magnetic susceptibility of Fe_2_O_3_-PS4-MNP is consistent with the XRD data and confirms the magnetic characteristics of the material. Such values make this a material with attractive feature for magnetic separation (Table 4) [40].

**Table 4:** Magnetic susceptibility of the Fe_2_O_3_-PS4-MNP nanohybrid material.

Catalyst	Milling time (min)	Magnetic susceptibility (10^−6^ m^3^ kg^−1^)

Fe_2_O_3_-PS4-MNP	15	420
	30	337

### Catalytic activity

The catalytic activity of these materials has been investigated in two reactions: 1) the oxidation of benzyl alcohol to benzaldehyde and the 2) alkylation of toluene with benzyl chloride.

**Scheme 1:**
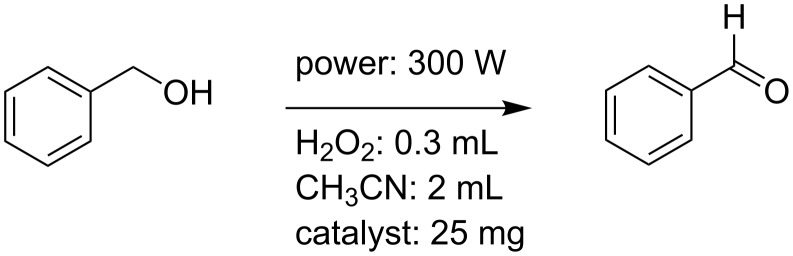
Oxidation of benzyl alcohol to benzaldehyde.

**Figure 5:**
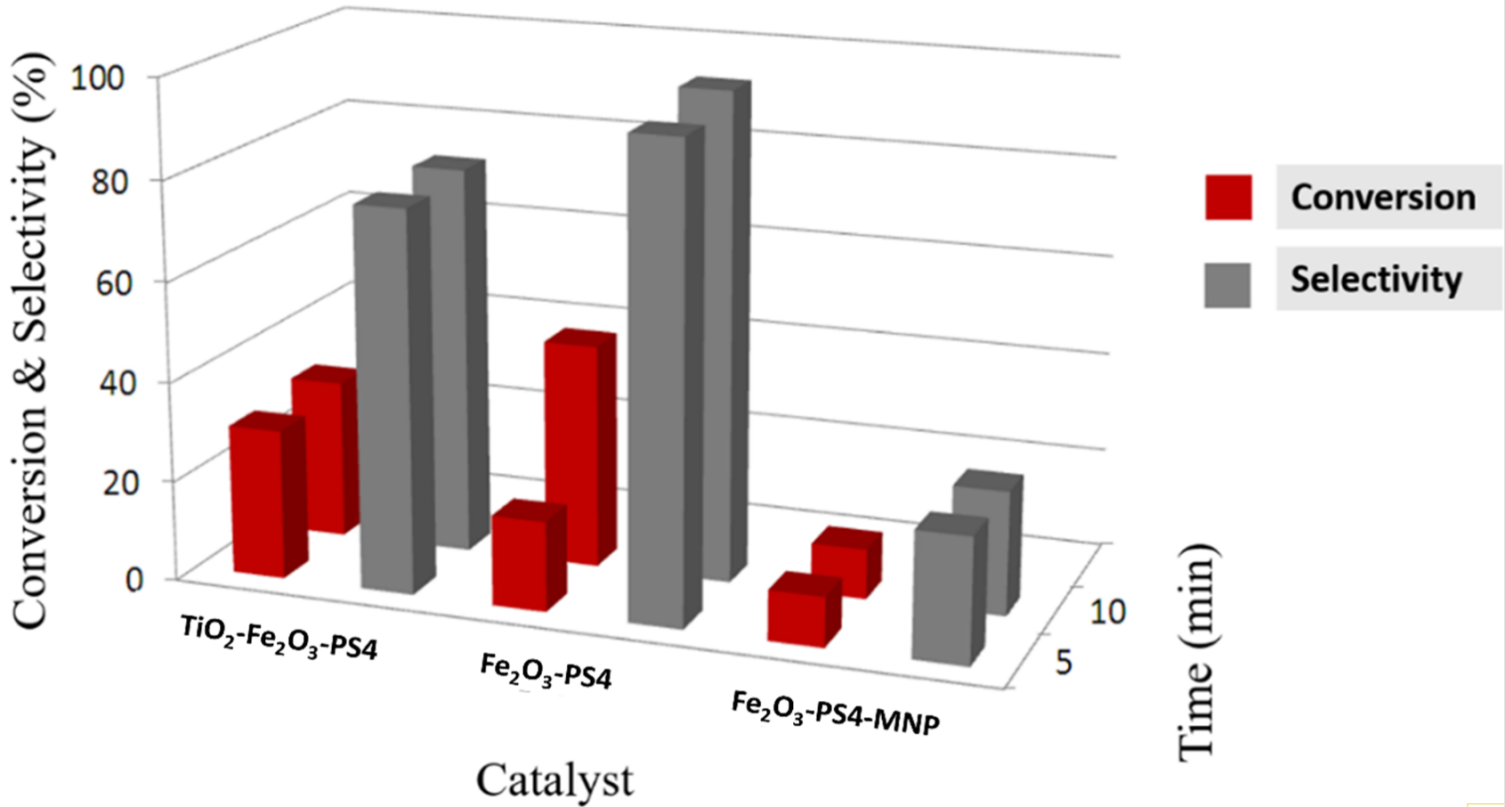
Conversion and selectivity of the oxidation of benzyl alcohol for the three catalytic systems.

The oxidation reaction of benzyl alcohol was carried out using the three nanomaterials as heterogeneous catalysts (Scheme 1). The results of conversion and selectivity are reported in Table 5 and Figure 5. After 10 min, the conversions were 32 and 45% for TiO_2_-Fe_2_O_3_-PS4 and Fe_2_O_3_-PS4 nanomaterials, respectively, while for the Fe_2_O_3_-PS4-MNP catalyst the conversion reaches just 10%. Remarkably, the selectivity to benzaldehyde, employing Fe_2_O_3_-PS4 nanocatalysts, was higher than 90% for a reaction time of 5 and 10 min. Since the best results were obtained with the Fe_2_O_3_-PS4 nanocomposite, the latter was employed to carry out the reaction for 30 min in order to improve the obtained results. However, the conversion increased only to 47%, which does not compensate the energy consumption by extending the reaction time from 10 to 30 min.

**Table 5:** Conversion and selectivity of the oxidation reaction of benzyl alcohol.

Catalyst	TiO_2_-Fe_2_O_3_-PS4		Fe_2_O_3_-PS4		Fe_2_O_3_-PS4-MNP	

Time (min)	C^a^ (%)	S^b^ (%)	C^a^ (%)	S^b^ (%)	C^a^ (%)	S^b^ (%)

5	30	76.6	18	94.4	10	24.4
10	32	78.1	45	97.7	10	24.7

^a^Conversion (%); ^b^selectivity (%) to benzaldehyde.

**Scheme 2:**
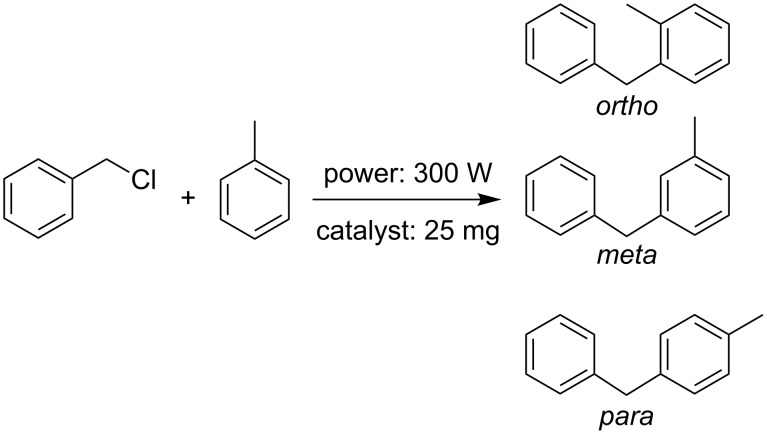
Microwave-assisted alkylation of toluene with benzyl chloride.

**Figure 6:**
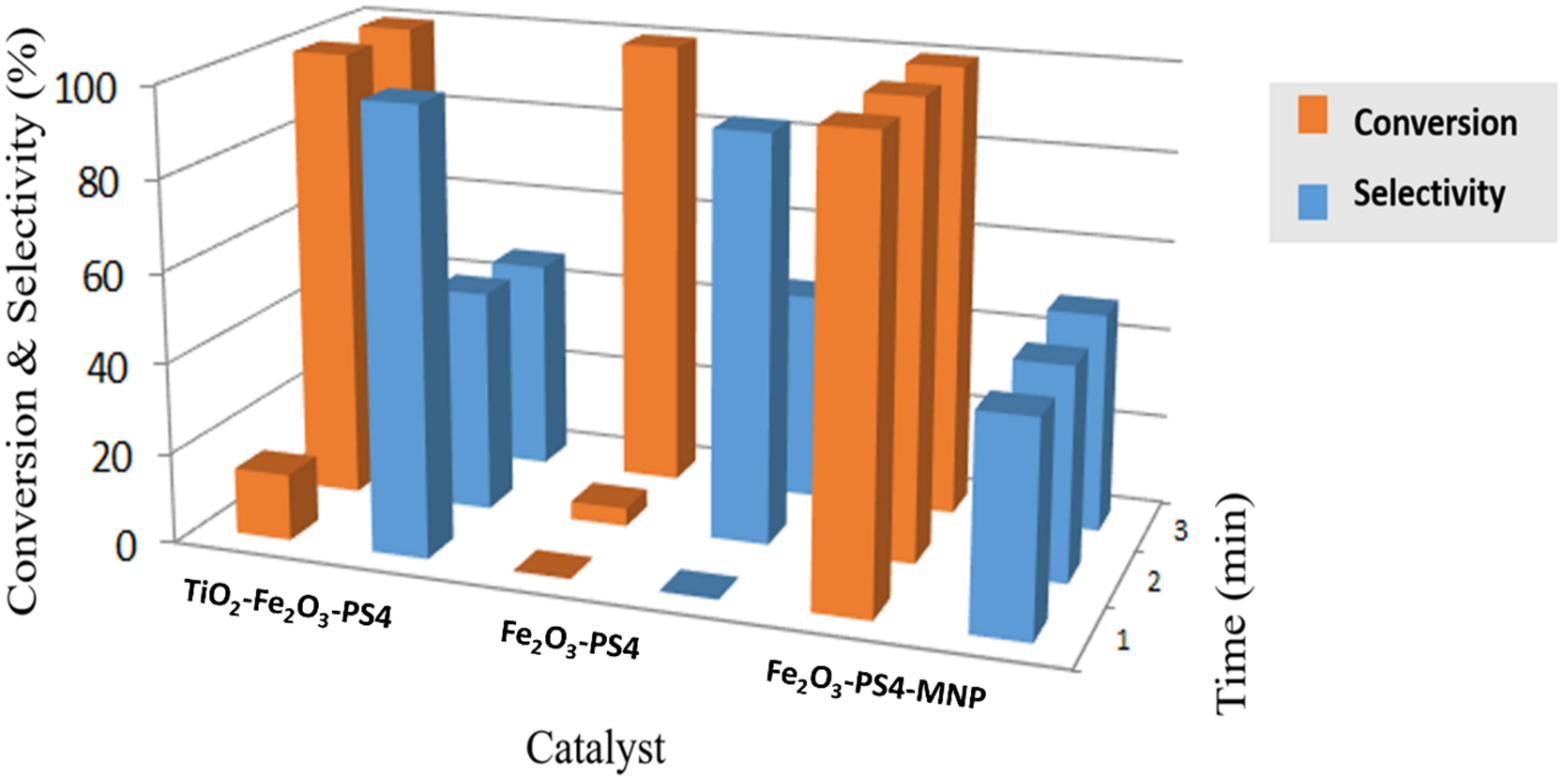
Conversion and selectivity of the microwave-assisted alkylation of toluene for the three catalytic systems.

The three synthesized catalysts showed high activity in the alkylation of toluene with benzyl chloride, either via microwave-assisted or with conventional heating (60 °C). For the microwave-assisted reaction (Scheme 2), after three minutes, the conversion was higher than 99% for all of the three materials (Figure 6). For the Fe_2_O_3_-PS4-MNP nanocatalyst, even after just 1 min, the reaction showed a conversion higher than 99%. In this reaction, the three corresponding isomers (*ortho*, *meta* and *para* substituted) were obtained. In particular, the synthesis of the *para*-isomer can be achieved with high selectivity, employing the TiO_2_-Fe_2_O_3_-PS4 and Fe_2_O_3_-PS4 nanomaterials for 1 and 2 min, respectively (Table 6).

**Table 6:** Conversion and selectivity of the microwave-assisted alkylation of toluene for the three catalytic systems.

Catalyst	TiO_2_-Fe_2_O_3_-PS4		Fe_2_O_3_-PS4		Fe_2_O_3_-PS4-MNP	

Time (min)	C^a^ (%)	S-p^b^ (%)	C^a^ (%)	S-p^b^ (%)	C^a^ (%)	S-p^b^ (%)

1	14.6	97.9	–	–	>99	46.3
2	>99	49.1	4	90.1	>99	46.9
3	>99	46.8	>99	46	>99	48.4

^a^Conversion (%); ^b^selectivity (%) with respect to the *para*-isomer.

**Scheme 3:**
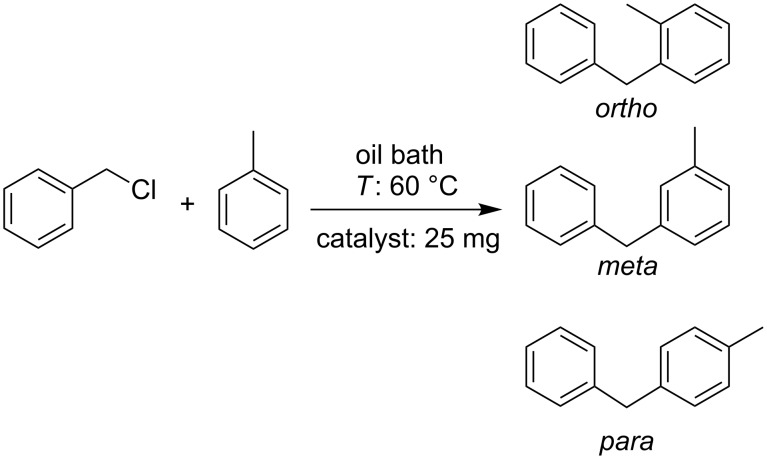
Alkylation of toluene with benzyl chloride with conventional heating.

**Figure 7:**
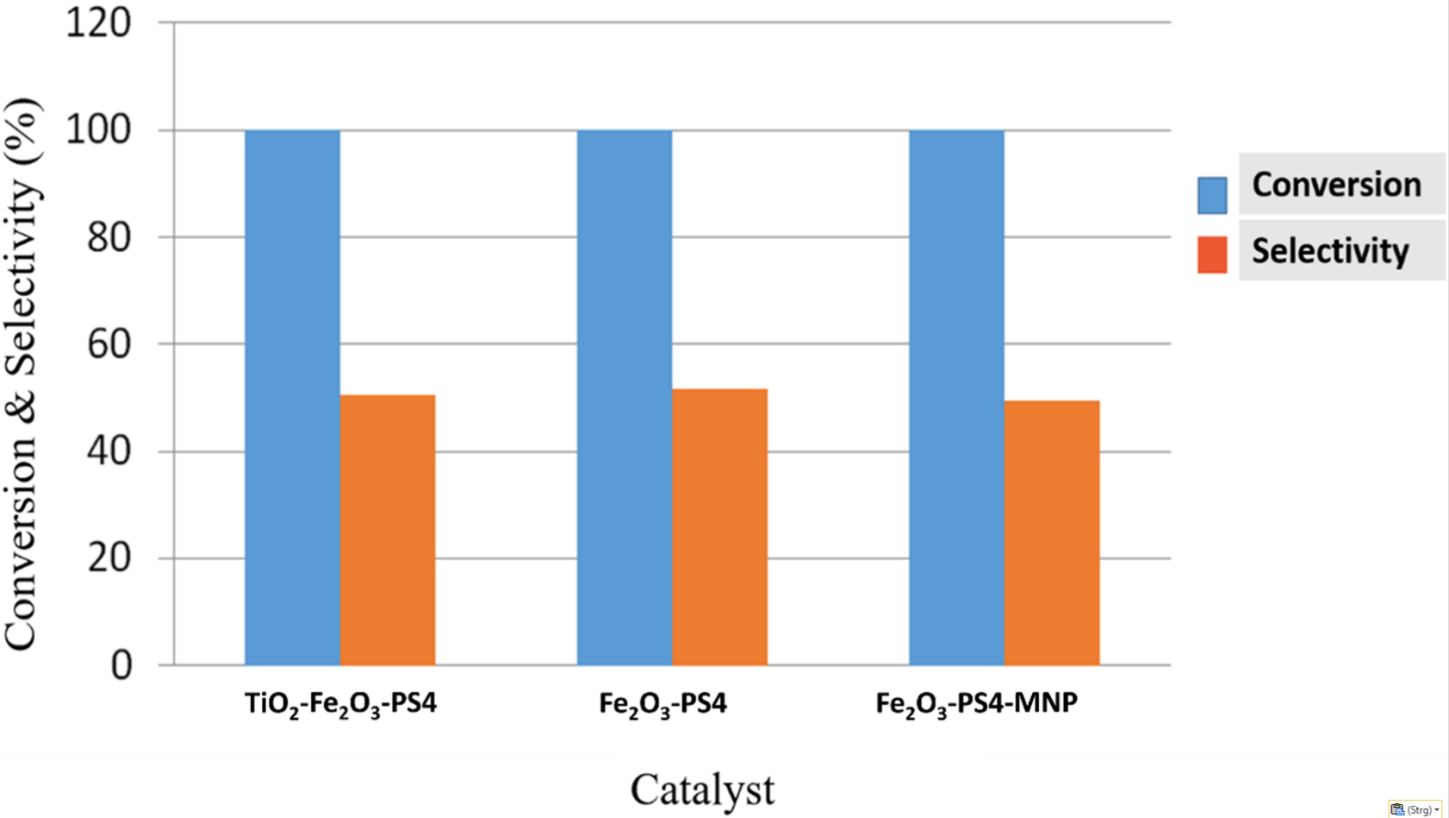
Conversion and selectivity of the alkylation of toluene with conventional heating for the three catalytic systems.

The alkylation reaction with conventional heating (Scheme 3) was followed by gas chromatography. After 30 min, the conversion was greater than 99% for all of the three materials and the selectivity values were slightly higher compared with the microwave-assisted reaction during 3 min (Table 7, Figure 7).

**Table 7:** Conversion and selectivity of the alkylation of toluene with conventional heating.

Catalyst	TiO_2_-Fe_2_O_3_-PS4		Fe_2_O_3_-PS4		Fe_2_O_3_-PS4-MNP	

Time (min)	C^a^ (%)	S-p^b^ (%)	C^a^ (%)	S-p^b^ (%)	C^a^ (%)	S-p^b^ (%)

30	>99	51.6	>99	50.5	>99	49.5

^a^Conversion (%), ^b^selectivity (%) with respect to the *para*-isomer.

**Figure 8:**
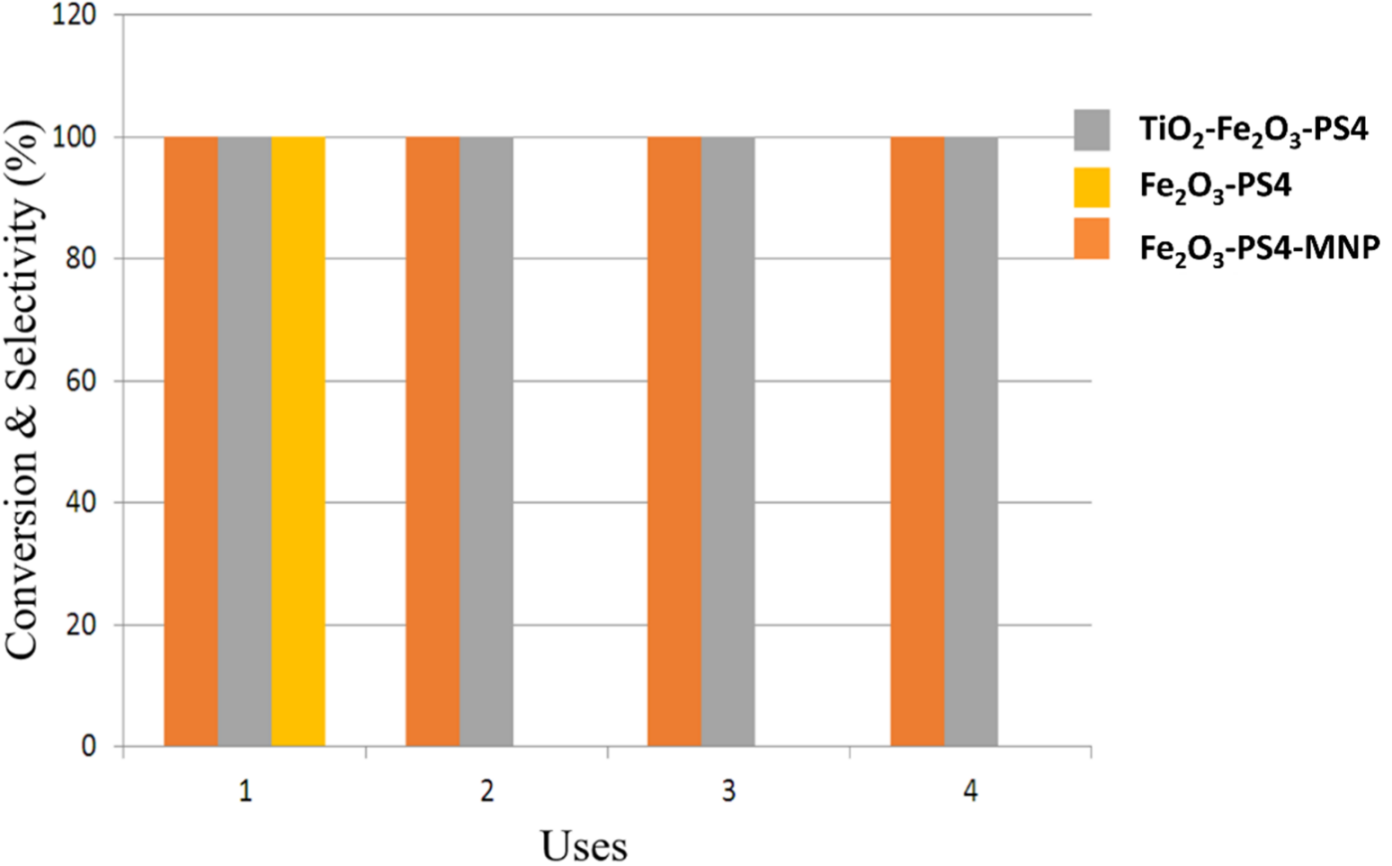
Reusability of the iron oxide/polysaccharide nanohybrids.

Reusability studies prove the high inherent stability and activity of Fe_2_O_3_-PS4-MNP and TiO_2_-Fe_2_O_3_-PS4 nanomaterials (Figure 8). However, the Fe_2_O_3_-PS4 nanocatalyst loses its activity after the first use, which can be due to the loss of residual acidity, which might happen to the material after the synthesis process.

Reference experiments for the two investigated reactions were carried out in the absence of catalyst, demonstrating that the nanocomposites play a crucial role in order to accelerate the reaction rates (Supporting Information File 1, Table S1). It can be concluded that for the oxidation of benzyl alcohol, the Fe_2_O_3_-PS4 presents a better employability outlook, whereas for the alkylation reaction, the Fe_2_O_3_-PS4-MNP showed promising conversion and selectivity values.

## Conclusion

The three bio-nanocomposites TiO_2_-Fe_2_O_3_-PS4, Fe_2_O_3_-PS4 and Fe_2_O_3_-PS4-MNP, based on iron oxide and polysaccharide S4 were synthesized by mechanochemical processes. The magnetic susceptibility measurements show attractive magnetic characteristics for recovery and reusability of the Fe_2_O_3_-PS4-MNP nanocomposite. Furthermore, the nanomaterials showed promising activity in the oxidation reaction of benzyl alcohol to benzaldehyde, with conversions of 32–45%. The three synthesized nanocomposites have proved to be highly active and selective catalysts in the alkylation reaction of toluene with benzyl chloride, due to the exceptional surface acidic properties of the nanoparticles. Both microwave irradiation and conventional heating exhibited high conversion and selectivity to the main product of the reaction in extremely short reaction times. Moreover, reusability studies showed high stability and activity of the nanohybrids TiO_2_-Fe_2_O_3_-PS4 and Fe_2_O_3_-PS4-MNP, establishing these catalysts as potential candidates in both the selective oxidation of benzyl alcohol and alkylation of toluene with benzyl chloride.

## Experimental

### Synthesis of bio-nanocomposites based on iron oxide and polysaccharide S4

A simple, reproducible and environmentally friendly protocol has been developed for the synthesis of the three catalysts Fe_2_O_3_-PS4, Fe_2_O_3_-PS4-MNP, and TiO_2_-Fe_2_O_3_-PS4. The three materials were synthesized using a 2:1 metal precursor/polysaccharide ratio (2 g of polysaccharide S4, 4 g of FeCl_2_·4H_2_O), in a ball mill (Retsch PM100 ball mill model), at 350 rpm for 30 min, using a 125 mL reaction chamber and 18 10 mm stainless steel balls. Additionally, in the case of the TiO_2_-Fe_2_O_3_-PS4, 4.08 mL of titanium isopropoxide was added to obtain the desired nanomaterial. Subsequently, the materials were oven-dried at 100 °C for 24 h, and finally calcined at 600 °C for 3 h in air atmosphere.

The Fe_2_O_3_-PS4-MNP catalyst was obtained using 2 g of polysaccharide S4, 4 g of Fe(NO_3_)_3_·9H_2_O and 1.5 mL of propionic acid. The milling process was carried out at 350 rpm for 15 and 30 min, respectively. The resulting material was oven-dried at 100 °C for 24 h and slowly heated up (1 °C/min) to 300 °C under air and kept at that temperature for 30 min.

### Material characterization

In order to characterize the synthesized materials, several techniques have been employed, including XRD, XPS, absorption–desorption of N_2_, SEM, TEM, DRIFT and titrations with pyridine and dimethylpyridine. In addition, the magnetic susceptibility of Fe_2_O_3_-PS4-MNP was measured and the elemental composition of TiO_2_-Fe_2_O_3_-PS4 was determined by ICP–MS.

#### X-ray diffraction

X-ray diffraction has been used for the structural study of the synthesized nanocatalysts. The diffraction patterns were obtained on a Bruker D8 Discover diffractometer, equipped with a goniometer Bragg Brentano θ/θ of high precision, and with a Cu X-ray tube. Scans were performed in the 0.5 to 80° range at a step size of 0.02° with a counting time per step of 20 s.

#### X-ray photoelectron spectroscopy

XPS measurements were performed at the Central Service of Research Support (SCAI) of the University of Cordoba, in an ultrahigh vacuum (UHV) multipurpose surface analysis system (SpecsTM model, Germany), operating at pressures of <10^−10^ mbar, using a conventional X-ray source (XR-50, Specs, Mg Kα, *h*v = 1253.6 eV, 1 eV = 1.603 × 10^−19^ J) in a "stop and go" mode. Powdered samples were deposited on a sample holder using double-sided adhesive tape and subsequently evacuated overnight under vacuum (<10^−6^ Torr). The spectra were taken at room temperature (pass energy: 25 and 10 eV, step size: 1 and 0.1 eV, respectively) with a Phoibos 150-MCD energy detector. For the deconvolution of the obtained curves, the XPS software CASA was used.

#### N_2_ physisorption

The Brunauer–Emmett–Teller (BET) surface area and pore volume measurements were obtained from N_2_ adsorption–desorption isotherms at liquid nitrogen temperature (77 K) in a Micromeritics ASAP 2000 instrument. The weight of the samples ranged between 0.15–0.20 g. Prior to the analysis, the samples were degassed for 24 h at 140 °C under vacuum (*p* < 10^−2^ Pa). The surface areas were calculated according to the linear equation of BET in the 0.05 < *p*_0_ < 0.22 range. The pore size distributions (PSDs) were obtained from the N_2_ desorption branch.

#### Electron microscopy

SEM images and the elemental composition were recorder using the JEOL JSM-6490 LV microscope. The samples were Au/Pd-coated on a high-resolution sputter SC7640 at a sputtering rate of 1.5 kV per minute, up to 7 nm thickness. TEM micrographs were obtained in the FEI Tecnai G^2^ system, equipped with a charge coupling device camera. Prior to analysis, the samples were suspended in ethanol and directly deposited on a copper grid.

#### Diffuse reflectance infrared Fourier transform spectroscopy

The DRIFT spectra of the materials were recorder on an infrared spectrophotometer (ABB MB3000 with Horizon MBTM software), equipped with an ATR PIKE MIRacleTM sampler, with a ZnSe window using 256 scans at a resolution of 8 cm^−1^. During the measurements, the sample was purged with a nitrogen flow (20 mL min^−1^, dehydrated and deoxygenated). The spectra were recorded at room temperature in the 4000–600 cm^−1^ wavenumber range. The materials were heated at 300 °C for 3 h prior to acquiring the reference spectra. Thus, the temperature was decreased to 200 °C, and after 10 min the reference spectrum was again recorded. Similarly, the reference spectra at 150 and 100 °C were acquired. Once the references were obtained, the acquisition of the spectra was carried out starting with the lowest temperature.

#### Pyridine (PY) and 2,6-dimethylpyridine (DMPY) titration

Pyridine (PY) and 2,6-dimethylpyridine (DMPY) titration experiments were carried out at 300 °C, via gas phase adsorption of the basic probe molecules applying a pulse chromatographic titration methodology. The catalyst used (≈0.025 g) was fixed inside a tubular stainless steel microreactor (4 mm internal diameter) by Pyrex glass wool. A cyclohexane solution of titrant (0.989 M in PY and 0.686 M in DMPY, respectively) was injected into a gas chromatograph through a microreactor in which the catalyst was previously sited. The injected base was analyzed by gas chromatography with a flame ionization detector and using an analytical column of 0.5 m length, containing 5 wt % of polyphenylether in the Chromosorb AW-DMCS in 80/100. The quantity of probe molecule adsorbed by the solid acid catalyst can subsequently be easily quantified. In order to distinguish between Lewis and Brønsted acidity, it was assumed that all DMPY selectively titrates Brønsted sites (methyl groups hinder coordination of nitrogen atoms with Lewis acid sites) while PY titrates both Brønsted and Lewis acid sites in the materials. Thus, the difference between the amounts of PY (total acidity) and DMPY (Brønsted acidity) adsorbed should correspond to Lewis acidity in the materials.

#### ICP–MS

The metal content in the TiO_2_-Fe_2_O_3_-PS4 catalyst was determined by ICP–MS in an Elan DRC-e (PerkinElmer SCIEX) spectrometer. The sample (≈25 mg) was previously digested using an acid mixture of HF/HNO_3_/HCl 1:1:1. Dilutions were made with miliQ water (double distilled) up to a maximum of 1% of HF_2_^−^ in acid solution.

#### Magnetic susceptibility

The magnetic susceptibility was measured at room temperature at low frequency (470 Hz) using a Bartington MS-2 instrument.

#### Catalytic experiments

The oxidation of benzyl alcohol to benzaldehyde was performed using 25 mg of catalyst, 0.2 mL of benzyl alcohol, 0.3 mL of hydrogen peroxide, and 2 mL of acetonitrile as the solvent, for 5 and 10 min, respectively.

The microwave-assisted alkylation of toluene and oxidation of benzyl alcohol was carried out in a CEM-Discover microwave reactor, equipped with a PC-controlled interface. The alkylation reactions were carried out by the standard "open vessel" method, while for oxidation reactions, the "discover" method was used under pressure, allowing us to control the irradiation power, temperature and pressure.

The alkylation reaction of toluene with benzyl chloride was performed under conventional heating, too. In both alkylation experiments, 2 mL of toluene, 0.2 mL of benzyl chloride and 25 mg of catalyst were used. The microwave-assisted reaction was conducted for 1, 2 and 3 min, while the reaction under conventional heating was carried out for 30 min until the maximum conversion was reached. The temperature in both cases was kept at around 60 °C.

The conversion and selectivity were calculated from the chromatograms by:


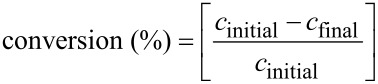



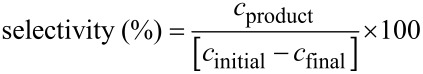


where *c*_initial_ and *c*_final_ are the concentrations of the reagents before and after the reaction, respectively, and *c*_product_ is the concentration of the product, as determined by gas chromatography (GC).

The samples were analyzed with a HP5890 Series II gas chromatograph (60 mL min^−1^ N_2_ carrier flow, 20 psi column top head pressure) using a flame ionization detector (FID). A HP-101 capillary column (25 m × 0.2 mm × 0.2 μm) was employed. All calculations were based on the use of benzyl chloride and benzyl alcohol as limiting reagents for the studied alkylation and oxidation reaction, respectively.

## Supporting Information

File 1 Additional spectra.
