# Supplementary material for: New bio-nanocomposites based on iron oxides and polysaccharides applied to oxidation and alkylation reactions
Source: Beilstein J Org Chem. 2017 Sep 21;13:1982–93. doi: 10.3762/bjoc.13.194 (PMC5629400; doi:10.3762/bjoc.13.194)
Supplement: File 1 — Additional spectra. [file Beilstein_J_Org_Chem-13-1982-s001.pdf]

Supporting Information

for

New bio-nanocomposites based on iron oxides and polysaccharides, applied to oxidation and alkylation reactions

Daily Rodríguez-Padrón, Alina M. Balu, Antonio A. Romero and Rafael Luque*

Address: Departamento de Química Orgánica, Grupo FQM-383, Universidad de Cordoba, Campus de Rabanales, Edificio Marie Curie (C-3), Ctra Nnal IV-A, Km 396, E14014, Cordoba, Spain

Email: Rafael Luque - q62alsor@uco.es

*Corresponding author

Additional spectra

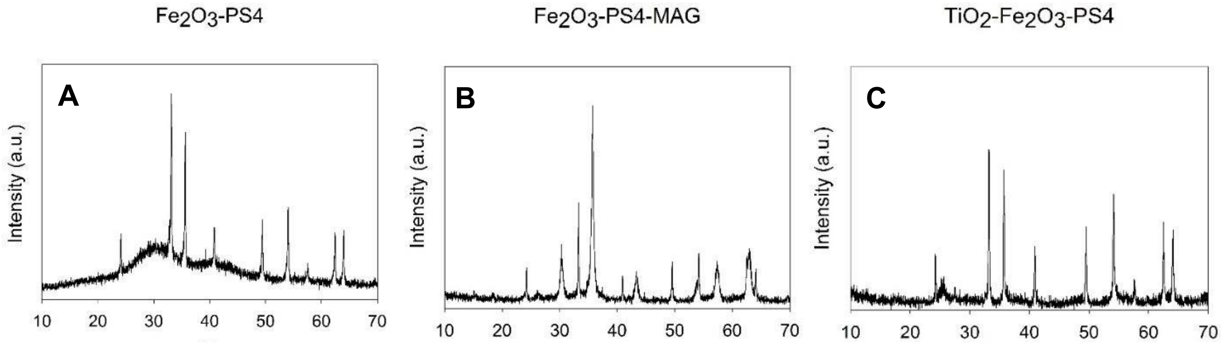

Figure S1. XRD patterns of A: Fe_2O_3-PS4, B: Fe_2O_3-PS4-MNP and C: TiO_2-Fe_2O_3-PS4 nanohybrids.

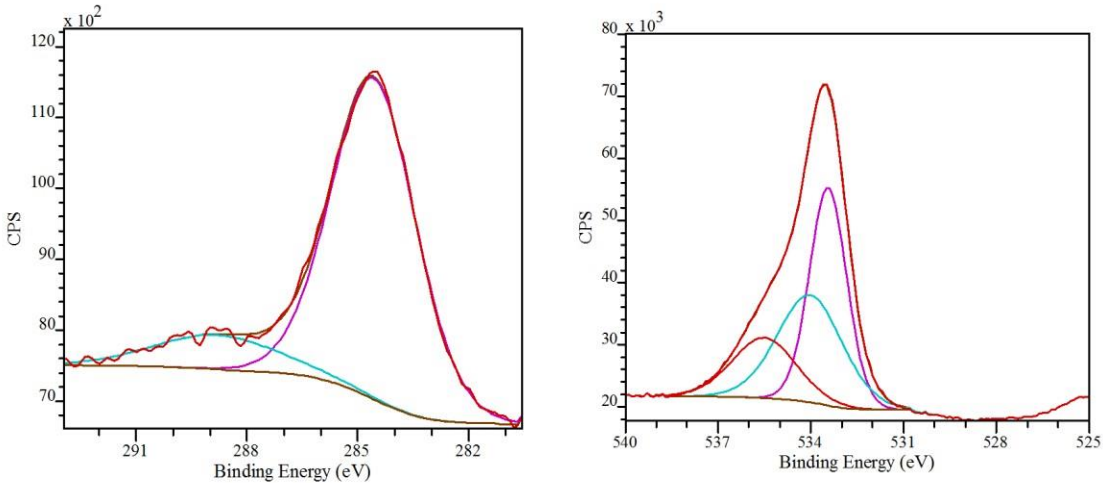

Figure S2. XPS spectra of TiO_2-Fe_2O_3-PS4 nanohybrid.

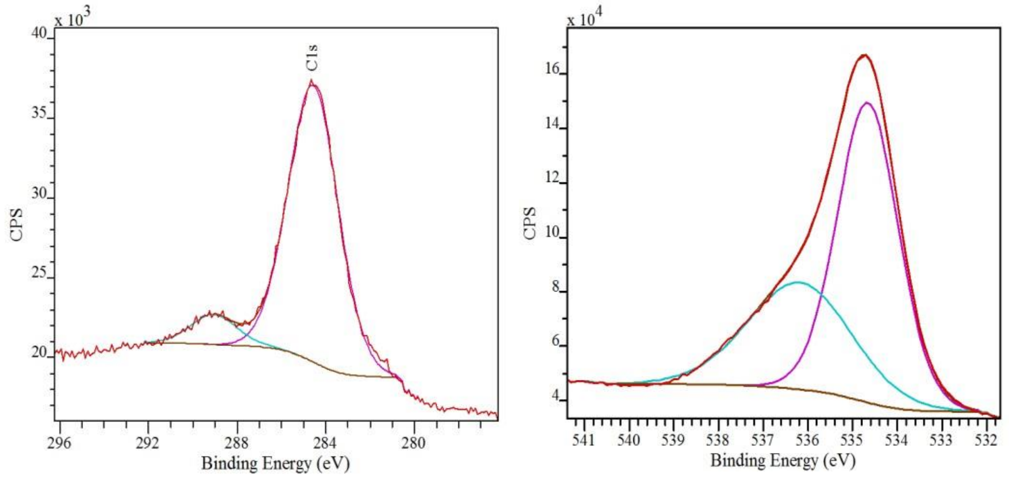

Figure S3. XPS spectra of Fe_2O_3-PS4-MNP nanohybrid.

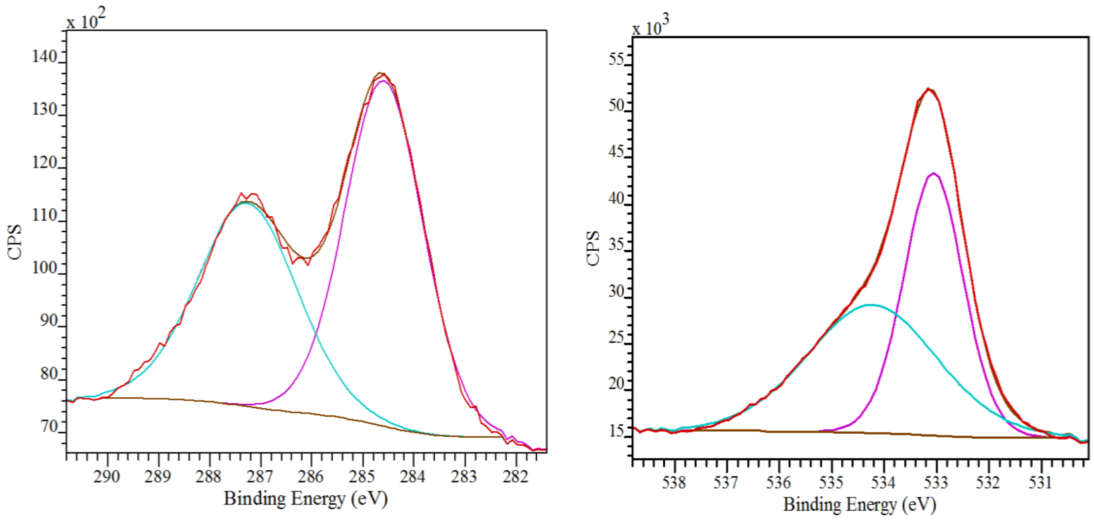

Figure S4. XPS spectra of Fe_2O_3-PS4 nanohybrid.

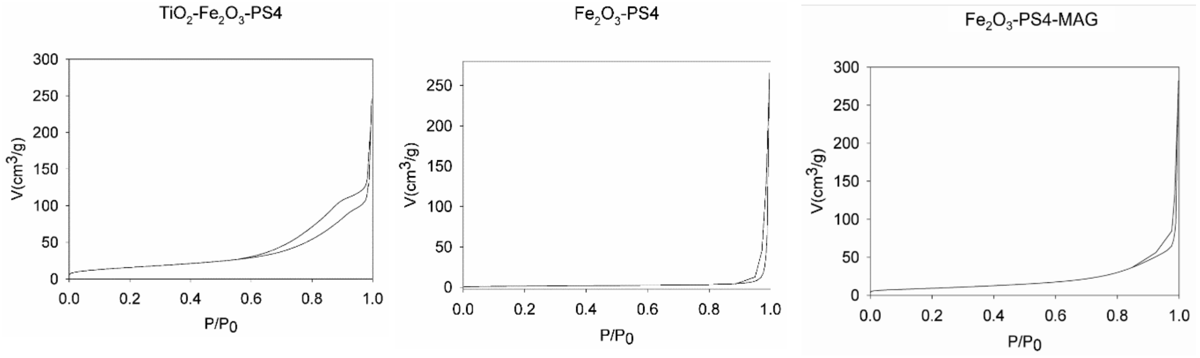

Figure S5. Adsorption-desorption isotherm of TiO_2-Fe_2O_3-PS4, Fe_2O_3-PS4 and Fe_2O_3- PS4-MNP nanohybrids.

Table S1: Blank experiments of the two investigated reactions: 1) alkylation of toluene and 2) oxidation of benzyl alcohol.

Reaction	Conversion (%)
Alkylation of toluene	<1
Oxidation of benzyl alcohol	<1
